# Hypomethylation of PlncRNA-1 promoter enhances bladder cancer progression through the miR-136-5p/Smad3 axis

**DOI:** 10.1038/s41419-020-03240-z

**Published:** 2020-12-07

**Authors:** Weiting Kang, Qiang Wang, Yun Dai, Hanbo Wang, Muwen Wang, Jin Wang, Dong Zhang, Peng Sun, Taiguo Qi, Xunbo Jin, Zilian Cui

**Affiliations:** 1 Department of Urology, Shandong Provincial Hospital, Cheeloo College of Medicine, Shandong University, Jinan, Shandong 250021 China; 2 Department of Human Resources, Shandong Provincial Hospital Affiliated to Shandong First Medical University, Jinan, Shandong 250021 China; 3 Department of Human Resources, Shandong Provincial Hospital, Cheeloo College of Medicine, Shandong University, Jinan, Shandong 250021 China; 4 Department of Ultrasound, Shandong Provincial Hospital Affiliated to Shandong First Medical University, Jinan, Shandong 250021 China; 5 Department of Ultrasound, Shandong Provincial Hospital, Cheeloo College of Medicine, Shandong University, Jinan, Shandong 250021 China; 6 Department of Urology, Shandong Provincial Hospital Affiliated to Shandong First Medical University, Jinan, Shandong 250021 China; 7 Department of Urology, The First Affiliated Hospital of Shandong First Medical University, Jinan, Shandong 250021 China; 8 Department of Urology, Shandong Provincial Qianfoshan Hospital, Cheeloo College of Medicine, Shandong University, Jinan, Shandong 250021 China

**Keywords:** Bladder cancer, Long non-coding RNAs, Oncogenesis

## Abstract

Apart from being potential prognostic biomarkers and therapeutic targets, long non-coding RNAs (lncRNAs) modulate the development and progression of multiple cancers. PlncRNA-1 is a newly discovered lncRNA that exhibits the above properties through multiple regulatory pathways. However, the clinical significance and molecular mechanisms of PlncRNA-1 in bladder cancer have not been established. PlncRNA-1 was found to be overexpressed in 71.43% of bladder cancer tissues. Moreover, the expression level correlated with tumor invasion, T stage, age, and number of tumors, but not with gender, recurrent status, preoperative treatment, pathological grade, and tumor size. The expression level of PlncRNA-1 can, to a certain extent, be used as a predictor of the degree of tumor invasion and T stage among BC patients. Inhibiting PlncRNA-1 expression impaired the proliferation, migration, and invasion of T24 and 5637 bladder cancer cells in vitro and in vivo. Specifically, PlncRNA-1 promoter in BC tissues was found to be hypomethylated at position 131 (36157603 on chromosome 21). PlncRNA-1 promoter hypomethylation induces the overexpression of PlncRNA-1. In addition, PlncRNA-1 modulated the expression of smad3 and has-miR-136-5p (miR-136). Conversely, miR-136 regulated the expression of PlncRNA-1 and smad3. PlncRNA-1 mimics competitive endogenous RNA (ceRNA) in its regulation of smad3 expression by binding miR-136. Rescue analysis further revealed that modulation of miR-136 could reverse the expression of smad3 and epithelial–mesenchymal transition (EMT) marker proteins impaired by PlncRNA-1. In summary, PlncRNA-1 has important clinical predictive values and is involved in the post-transcriptional regulation of smad3. The PlncRNA-1/miR-136/smad3 axis provides insights into the regulatory mechanism of BC, thus may serve as a potential therapeutic target and prognostic biomarker for cancer.

## Introduction

Bladder cancer is one of the most common malignant tumors of the urogenital system. Globally, more than 430,000 individuals are annually diagnosed with bladder cancer, with nearly 170,000 mortality cases^1,2^. Depending on the degree of muscle invasion, bladder cancer is divided into nonmuscle invasive bladder cancer (NMIBC) and muscle-invasive bladder cancer (MIBC) types. NMIBC accounts for about 70% of newly diagnosed BC and can be treated by transurethral resection and invasive therapy. However, it often relapses and develops into MIBC^2,3^. MIBC is highly metastatic and has poor prognostic outcomes^4^. Due to its metastatic nature, the 5-year overall survival rate of MIBC patients is 60% after surgical resection and chemotherapy^5^. Therefore, elucidating the molecular mechanisms involved in bladder cancer pathogenesis, potential early biomarkers together with more effective and safer therapeutic options can significantly improve BC prognosis.

LncRNA is a large (more than 200 nucleotides) noncoding RNA that performs multiple functions. It is involved in multiple cellular processes, including chromatin remodeling, transcription and translation regulation, RNA stability, scaffold, and innate immunity^6^. It is also central in the regulation of gene expression in many diseases^7^. LncRNA-BLACAT2, lncRNA-LET, LncRNA-SLC16A1-AS1, SOX2OT, and LNMAT2 are involved in different key roles in the occurrence and development of bladder cancer^8–12^. Mechanistically, lncRNAs perform their regulatory functions by acting as ceRNAs against the expression of multiple genes. LncRNAs exhibit sequence similarities with several protein transcripts, thus confer their functions by competing with miRNA regulatory molecules^13^. For instance, lncRNA DANCR is significantly upregulated in bladder cancer and positively regulates the expression of MSI2 through miR-149 sponging. This in turn enhances the pathogenesis and malignancy of bladder cancer^14^.

Overexpressed lncRNA PlncRNA-1 (transcript Variant 3 of CBR3-AS 1) promotes the proliferation and apoptosis of prostate cancer cells^15^. Androgen receptor (AR) promotes PlncRNA-1 expression. Furthermore, PlncRNA-1 mediates microRNA sponging, targeting AR, a process that protects AR from microRNA-mediated downregulation. This generates a regulatory feed-forward loop in the development of prostate cancer^16^. Studies have also revealed that differential PlncRNA-1 expression in multiple diseases is involved in; modulation of cell proliferation, apoptosis, metastasis, epithelial–mesenchymal transition (EMT), and autophagy^17–29^. However, the clinical significance and biological functions of PlncRNA-1 in bladder cancer have not been established.

In this study, we elucidated the expression changes and biological functions of PlncRNA-1 in bladder cancer and its clinical significance. In particular, hypo DNA methylation of the PlncRNA-1 promoter region induces its overexpression. On the other hand, the expression level of PlncRNA-1 corresponds with the clinical stage of BC. PlncRNA-1 plays an important role in the proliferation and invasion of bladder cancer cells in vitro and vivo. Mechanistic studies revealed that PlncRNA-1 can induce miR-136 sponging in a ceRNA like a manner, positively regulating the expression of smad3. In summary, our results reveal that PlncRNA-1 is a strong tumor biomarker and a promising therapeutic and diagnostic target for BC.

## Materials and methods

### Bladder cancer samples

From May 2014 to June 2020, 28 pairs of bladder cancer tissues, and normal matched tissues were collected from patients attending Shandong Provincial Hospital, Department of Urology. Among them, 18 and 10 samples were extracted from primary and recurrent BC tissues, respectively. To be included in the study, the participants must have been pathologically diagnosed with bladder cancer. All the 28 paired samples were subjected to quantitative polymerase chain reaction (qPCR) analysis, whereas only 6 of them were randomly selected, using random number tables, for methylation analysis. All study participants consented to the study in writing and by signing a consent form. Ethical approval was obtained from the Ethics Committee of Shandong Provincial Hospital (Jinan, China).

### Cell lines and cell culture

Human BC cell lines (T24, 5637, J82, and RT4) were obtained from the Cell Bank of the Chinese Academy of Sciences (Shanghai, China). T24 and RT4 cells were cultured in McCoy’s 5A medium (Invitrogen, CA, USA), 5637 cells were cultured in RPMI-1640 medium (Gibco, CA, USA) while J82 cells were cultured in minimal essential medium (Sigma, MO, USA). The media were supplemented with 10% fetal bovine serum (FBS) (Gibco, CA, USA) and 100 IU/ml penicillin (Sigma). The cell cultures were incubated at 37 °C under 5% CO_2_. All cell lines were mycoplasma free.

### RNA extraction and quantitative real-time PCR

Total RNA was extracted from BC cells using the RNAiso Plus kit (TAKARA) while miRNA was extracted using the RNAiso kit (TAKARA, Japan) according to the manufacturers’ instructions. Complementary DNA (cDNA) for the extracted total RNA was synthesized by reverse transcription of the total RNA using PrimeScript RT Reagent kit (Takara), according to the manufacturers’ instructions. cDNA for miRNA was synthesized using the Mix-X miRNA First-Strand Synthesis Kit (Takara), according to the manufacturers’ instructions. The qPCRs were performed using SYBR Premix Ex Tap (Takara) and amplified in LightCycler 480II (Roche, Switzerland). The relative expression of genes was calculated using the 2^−ΔΔCt^ method.

### Cell transfection

The siRNA and siRNA negative controls against PlncRNA-1 were purchased from Sangon Biotech (Shanghai, China) while MiR-136-5p mimic, miRNA mimic control, miR-34a-5p inhibitor, and miRNA inhibitor controls were purchased from Genomeditech (Shanghai, China). Cell transfection using lipofectamine 3000 (Invitrogen) was performed following the manufacturer’s protocol. The same concentrations of negative siRNA control, miRNA mimic control, and miRNA inhibitor control were used in each transfection experiment. All transfection experiments were performed within 48 h. qPCR analysis was used to detect the level of gene expression and to assess the efficiency of silencing or over-expression of relevant genes.

### Cell viability, clonability, migratory, and invasion tests

Cell viability was measured at 0, 24, 48, 72, and 96 h using the Cell Counting Kit 8 (Dojindo, Japan) according to the manufacturer’s instructions. The spectrophotometric absorbance of each sample at 450 nm was assessed using a spectrophotometer (Multiskan Go, Thermo Fisher Scientific, Finland). To assess colony formation, low cell densities (500 cells/plate) were seeded in 6-well plates 48 h after transfection and allowed to form visible colonies. The colonies were then counted after staining with crystal violet. Migration of bladder cancer cells was assessed using a wound-healing assay. Briefly, after reaching 90% cell confluence, the transfected cells were scraped off the culture plate using a 200 µl sterile pipette tip. Floating cells were washed off. The size of the wound was measured and photographed at 0, 24, and 48 h. For invasion tests, 5 × 10^4^ transfected bladder cancer cells in serum-free medium were seeded both in uncoated and Matrigel-coated (BD Bioscience, CA, USA) upper chambers of the well plates (Costar, NY, USA). Cells in the lower wells were supplemented with a culture medium containing 10% FBS and, thereafter, cultivated for a further 24 h. Cell counting during the assessment of migration and invasion was performed in three random fields.

### Nude mouse xenograft model

Protocols for animal experiments were approved by the Institutional Animal Care Committee with manipulations performed in line with laid down ethical regulations by the Committee of Shandong Provincial Hospital. For tumor xenograft establishment, ten nude mice were randomly allocated into two groups by a random number table. A suspension of 5637 single-cells (1 × 10^7^ cells/0.1 µm) was subcutaneously injected into 4-week-old male BALB/c nude mice (Vital River, Beijing, China). The length (*L*) and width (*W*) of the subcutaneous xenograft were measured blindly and its height (*H*) estimated after every 3 days. Tumor volumes were calculated according to the formula: *V* = *π*/6*(L*W*H), from which growth curves for the tumors were plotted. Tumors were extracted, weighed, and photographed 4 weeks later from killed mice.

### DNA methylation analysis

The methylation level of the PlncRNA-1 promoter in cancer and normal tissues was validated using the Wanderer online tool (http://maplab.imppc.org/wanderer/)^30^. Thereafter, MEXPRESS (https://mexpress.be/index.html) was used to determine the correlation between the degree of PlncRNA-1 promoter methylation and the corresponding expression level of PlncRNA-1 in bladder cancer cells^31,32^. BC T24 and 5637 cell lines were treated with different concentrations of 5-aza-2′-deoxycytidine (AZA) (Sigma). The DNA from bladder cancer cells was extracted using the QIAamp DNA Mini Kit (Qiagen, Germany), according to the manufacturer’s instructions. DNA methylation using sodium bisulphite was performed using the EZ DNA methylation-Gold kit (Zymo Research, CA, USA), according to the manufacturer’s instructions. Bisulfite-treated DNA was amplified or cloned by methylation-specific quantitative amplification of the methylated and unmethylated PlncRNA-1 promoter regions. Sequencing of the amplicons was performed at Sangon Biotech. BSAS was performed by Genechem (Shanghai, China).

### Western blotting analysis

Total cell protein was extracted using RIPA Lysis Buffer (Beyotime, Shanghai, China), supplemented with protease and phosphatase inhibitors (APExBIO, USA). Protein concentration was determined using the Enhanced BCA Protein Assay Kit (Beyotime) according to the manufacturers’ instructions. Briefly, equal amounts of protein were first separated by sodium dodecyl sulphate-polyacrylamide gel electrophoresis and, thereafter, transferred to polyvinylidene difluoride membranes (Millipore). After 1 h of blocking with 5% skim milk, the membranes were incubated overnight at 4 °C with primary antibodies. After being washed thrice using TBST, the membranes were re-incubated for 1 h at room temperature with horseradish peroxidase-conjugated secondary antibodies. Protein bands were then visualized using an enhanced chemiluminescence system (Millipore, Bedford, MA) and subsequently analyzed using an Amersham image 800 (GE). GAPDH was used as the loading control. Antibodies against smad3 (ab40854), p-smad3 (ab52903), E-cadherin (ab1416), and vimentin (ab92547) were purchased from Abcam (MA, USA), whereas those against N-cadherin (13116) and GADPH (5174) were purchased from Cell Signaling Technology (MA, USA).

### RNA in situ fluorescent hybridization

PlncRNA-1 FISH probes were designed and synthesized by the RiboBio Company (Guangzhou, China). BC cells were collected after 48 h of transfection and subsequently seeded on glass coverslips. RNA FISH was performed using the fluorescent in situ hybridization kit (RiboBio), following the manufacturer’s instructions. Finally, the effulgent were visualized using a fluorescence scanning microscope (Leica, Germany).

### Dual-luciferase reporter assay

PlncRNA-1 wild type (WT), PlncRNA-1 mutation type 1 (MT 1), and PlncRNA-1 mutation type 2 (MT 2) were constructed by Genomeditech (Shanghai, China) and co-transfected into 293T cells along with the miR-136-5p mimic or miRNA controls using the Lipofectamine 3000 kit (Invitrogen). The cells were incubated for 48 h after transfection. Dual-luciferase reporter assays were performed using the Dual-Luciferase Reporter Assay System (Promega, WI, USA), following the manufacturer’s instructions. Luciferase functions were measured using Centro XS³ LB 960 Microplate Luminometer (Berthold Technologies, USA), following normalization by renilla luciferase.

### miRNA-seq

miRNA of bladder cancer 5637 cell line was sequenced 48 h after transfection at Sangon (Shanghai, China). miRNA target gene binding sites were predicted using Miranda software and starBase v 2.0^33^.

### Immunohistochemistry (IHC) of bladder cancer tissues

Tissue sections were embedded in paraffin and sectioned. To determine the protein expression level, IHC was performed based on the SP Link Detection kit (Rabbit Biotin-streptavidin HRP Detection Systems) (ZSGB-Bio, Beijing, China) following the manufacturer’s instructions. Tissue sections were then visualized using TissueFAXS (Vienna, Austria).

### Statistical analysis

Statistical analyses were performed using R software V 3.6.1. Results are presented as the mean ± standard error. Statistical differences between two groups were determined using the Student’s *t* test, whereas ANOVA was used to determine the statistical differences between three or more groups based on one factor. The strength of association between ranked variables was assessed using Spearman’s rank correlation test and *χ*^2^ test. Each experiment was replicated three times. All tests were two-sided. *p* ≤ 0.05 was considered to be statistically significant.

## Results

### PlncRNA-1 is highly expressed in bladder cancer

**Fig. 1:**
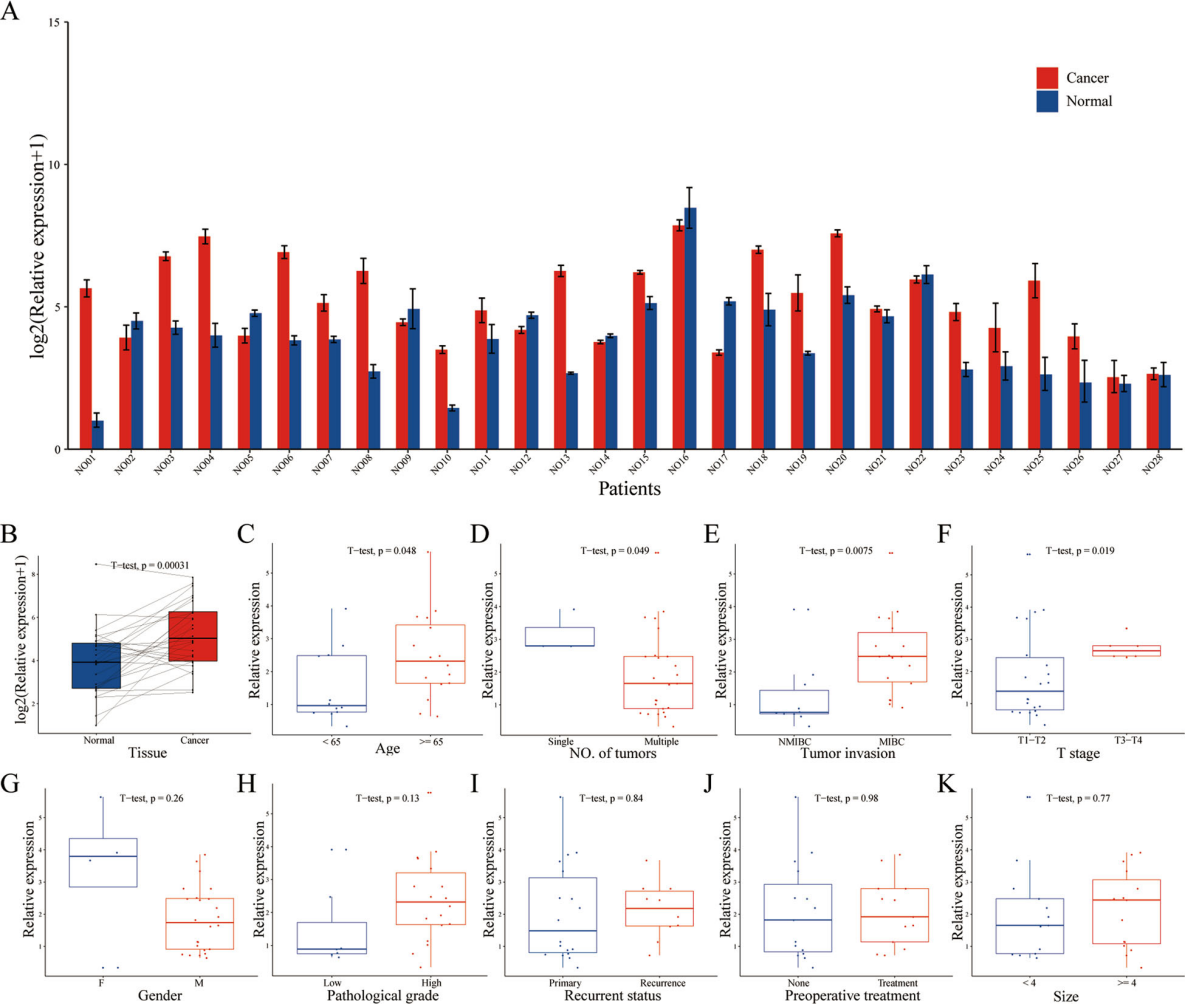
PlncRNA-1 expression levels in BC tissues. **A**, **B** The expression level of PlncRNA-1 in 28 pairs of normal and BC tissues. **C**–**K** The correlation between BC tissue-PlncRNA-1 expression and age, number of tumors, tumor invasion, T stage, gender, pathological grade, recurrent status, preoperative treatment, and tumor size.

Compared to the matched normal tissue, PlncRNA-1 was found to be significantly over-expressed in 71.43% (20/28) of the bladder cancer tissues (Fig. 1A, B). The relationship between the expression level of PlncRNA-1 and the corresponding clinicopathological characteristics of BC patients is shown in Fig. 1C–K. Compared to the age <65-year group, multiple tumor group, NMIBC group, and T1–T2 group, PlncRNA-1 was shown to be highly expressed in the age ≥65-year group (*p* = 0.048) (Fig. 1C), single tumor group (*p* = 0.049) (Fig. 1D), MIBC group (*p* = 0.0075) (Fig. 1E), and T3–T4 group (*p* = 0.019) (Fig. 1F), respectively. The expression level of PlncRNA-1 was not correlated with gender (*p* = 0.26) (Fig. 1G), pathological grade (*p* = 0.13) (Fig. 1H), recurrent status (*p* = 0.84) (Fig. 1I), preoperative treatment (*p* = 0.98) (Fig. 1J), and tumor size (*p* = 0.77) (Fig. 1K). Table 1 shows the correlation between the expression levels of PlncRNA-1 and clinicopathological features of BC patients. According to the median PlncRNA-1 expression level of 2.66, BC was divided into PlncRNA-1 high expression group and low expression group. In order to compare the predictive ability of the expression level of PlncRNA-1 on the clinical characteristics of patients with bladder cancer, we analyzed the proportions of different subgroups in the high or low expression group of PlncRNA-1. As shown in Table 1, in the low expression group, the T1–T2 stage accounted for 100% (14/14) while the MIBC stage accounted for 42.86% (6/14). In the high expression group, T1–T2 stage accounted for 57.14% (8/14), while the MIBC stage accounted for 85.71% (12/14). Therefore, according to the expression levels of PlncRNA-1, the degree of tumor invasion and T stage of BC patients can be predicted to a certain level.

**Table 1:** The correlation between the expression levels of PlncRNA-1 and clinicopathological features of BC patients.

Characteristics	Total	PlncRNA-1		*p* Value
		Low	High	
*Gender*				
M	24	13	11	0.596
F	4	1	3	
*Age (year)*				
<65	12	8	4	0.252
≥65	16	6	10	
*Recurrent status*				
Primary	18	10	8	0.693
Recurrence	10	4	6	
*Preoperative treatment*				
None	15	8	7	1.000
Treatment	13	6	7	
*Surgical approach*				
Partial cystectomy	3	1	2	1.000
Radical cystectomy	25	13	12	
*Pathological grade*				
Low	7	5	2	0.379
High	18	7	11	
NA	3	2	1	
*Size (cm)*				
<4 cm	13	7	6	1.000
≥4 cm	15	7	8	
*No. of tumors*				
Single	3	0	3	0.222
Multiple	25	14	11	
*Tumor invasion*				
NMIBC	10	8	2	0.049
MIBC	18	6	12	
*T stage*				
T1–T2	22	14	8	0.002
T3–T4	6	0	6	
*N stage*				
N0	25	14	11	0.222
N1	3	0	3	
*M stage*				
M0	27	14	13	1.000
M1	1	0	1	

### PlncRNA-1 modulates the proliferation, migration, and invasion of bladder cancer cells

**Fig. 2:**
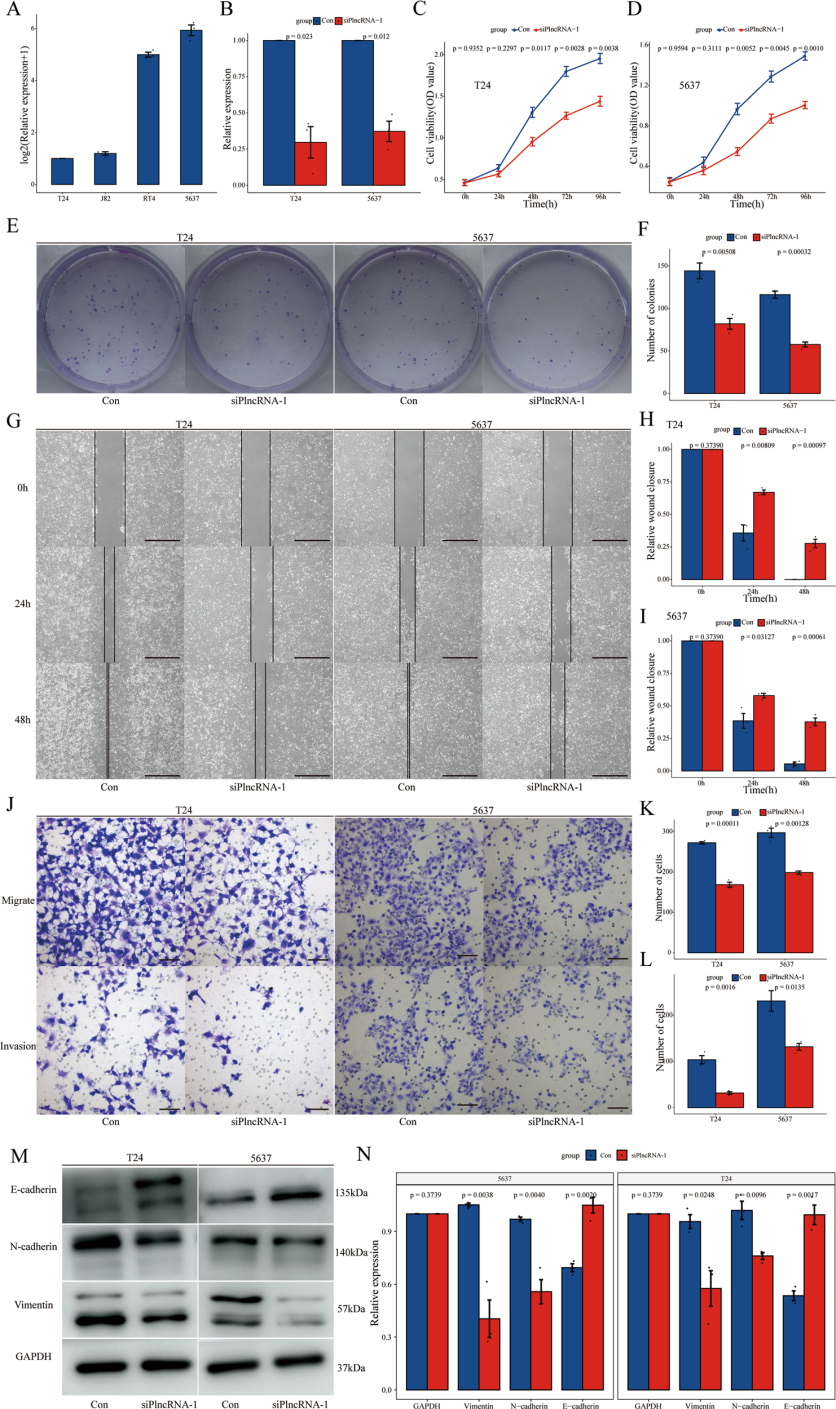
Effect of PlncRNA-1 knockdown on the proliferation, migration, and invasion of BC cells. **A** PlncRNA-1 expression levels in T24, J82, RT4, and 5637 BC cells. **B** qPCR analysis for the transfection efficiency of PlncRNA-1-siRNA in T24 and 5637 BC cells. **C**, **D**. CCK-8 assay for the proliferation of T24 and 5637 cells after PlncRNA-1 silencing. **E**, **F** Colony formation assay for the proliferative capacity of T24 and 5637 BC cell lines after PlncRNA-1 silencing. **G**–**I**. Wound healing assays for the migration of T24 and 5637 BC cells after PlncRNA-1 silencing (Scale bar, 200 μm). **J**–**L** Transwell migration and invasion assays for the migration and invasion of T24 and 5637 after PlncRNA-1 silencing (Scale bar, 100 μm). **M**, **N** Western blot analysis of the impact of PlncRNA-1 on EMT progression.

To explore the in vitro effect of PlncRNA-1 on the biological function of bladder cancer cells, we first evaluated the expression level of PlncRNA-1 in different bladder cancer cell lines (T24, J82, RT4, and 5637). PlncRNA-1 was found to be highly expressed in the 5637 cell line and suppressed in the T24 cell line (Fig. 2A). Accordingly, we interfered with PlncRNA-1 expression in 5637 and T24 cell lines using siRNA. As shown in Fig. 2B, compared to the control group, siRNA significantly modulated the expression level of PlncRNA-1 in the experimental group both in T24 and 5637 cell lines.

In addition, we used the Cell Counting assay (CCK-8) to analyze the effect of PlncRNA-1 on the proliferation of bladder cancer cells. Inhibition of PlncRNA-1 significantly decreased the proliferative ability of both T24 and 5637 BC cell lines (Fig. 2C, D). Colony formation analysis further showed that PlncRNA-1 inhibition significantly decreased the number of colonies for T24 and 5637 (Fig. 2E). Moreover, wound healing on the migratory ability of bladder cancer cells further revealed that PlncRNA-1 inhibition significantly impaired the migration of T24 and 5637 BC cells at 24 and 48 h (Fig. 2G–I). Transwell experiments also revealed that interference with PlncRNA-1 expression significantly inhibited the migration and invasion of both T24 and 5637 BC cells (Fig. 2J–L). Western blot was, therefore, performed to validate the expression level of EMT marker proteins. Compared to the controls, the expression of N-cadherin and Vimentin proteins was found to be significantly low in the siPlncRNA-1 group, converse to E-cadherin which was significantly overexpressed (Fig. 2M, N). These findings imply that inhibition of PlncRNA-1 can significantly suppress the proliferation, migration, and invasion of bladder cancer cells in vitro.

### PlncRNA-1 regulates the tumorigenicity of bladder cancer cells in vivo

**Fig. 3:**
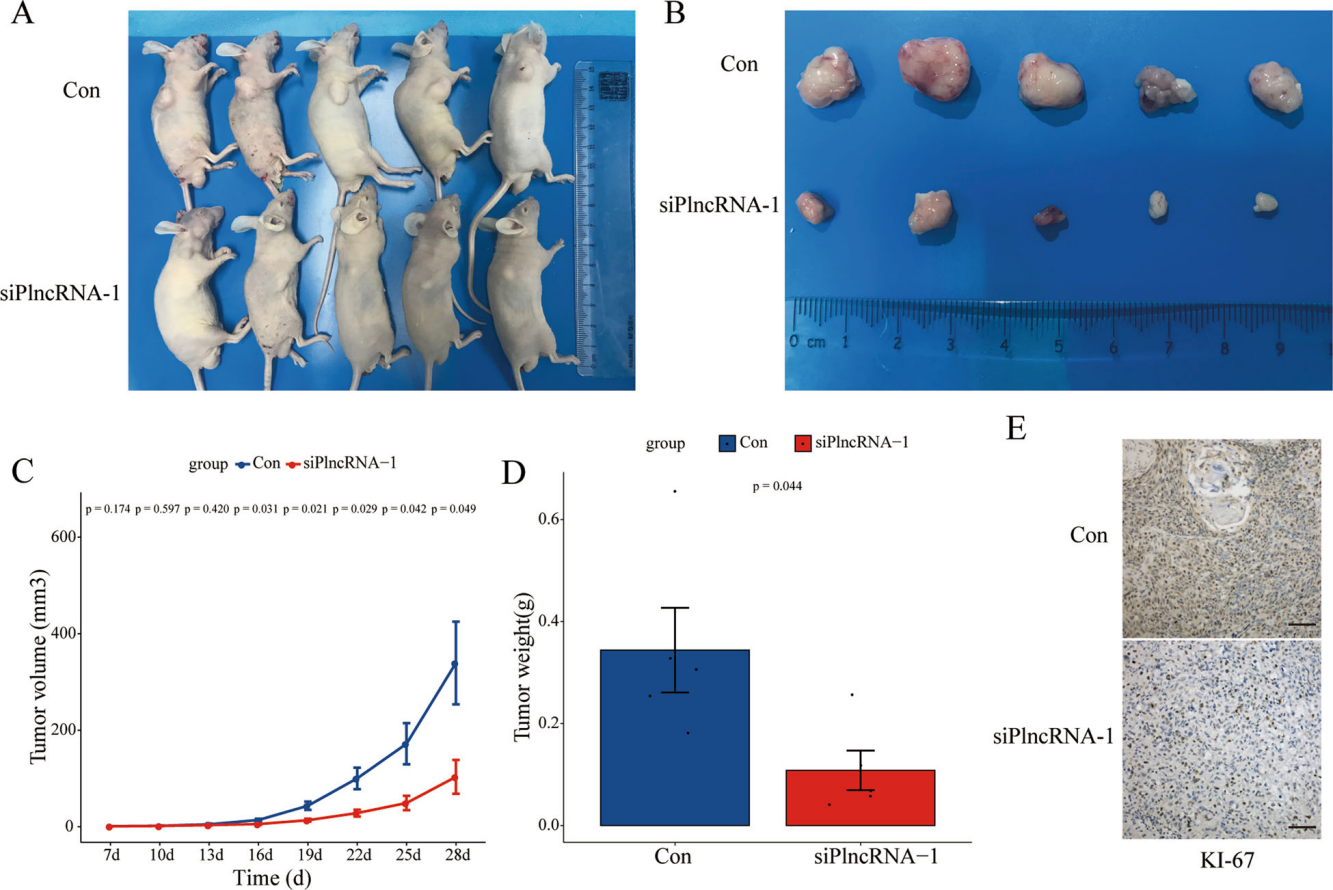
Xenograft tumor formation of 5647 cells in PlncRNA-1-silenced nude mice. **A** Photograph of xenografted tumors in nude mice on day 28. **B** Pictorials of subcutaneous tumors on day 28. **C** Growth curves for xenografted tumors. **D** The tumor weights of different groups. **E** Immunohistochemistry assay for the expression of Ki-67 in the tumor xenografts (Scale bar, 100 μm).

To study the carcinogenic ability of PlncRNA-1 to bladder cancer cells in vivo, 5636 BC cells that interfere with PlncRNA-1 expression were subcutaneously injected in male nude mice. After 4 weeks, inhibition of PlncRNA-1 expression significantly impaired tumor growth (Fig. 3A, B). In addition, the weight (Fig. 3C) and volume (Fig. 3D) of the implanted tumor were also significantly decreased in the PlncRNA-1 silenced group. Immunohistochemical staining analysis of the implanted tumor showed that inhibiting the expression of PlncRNA-1 significantly reduced the expression of Ki-67 which is closely associated with cell proliferation (Fig. 3E). These findings show that inhibiting the expression of PlncRNA-1 in vivo significantly impaired BC tumorigenicity.

### Hypomethylation of PlncRNA-1 promoter region promotes its expression

**Fig. 4:**
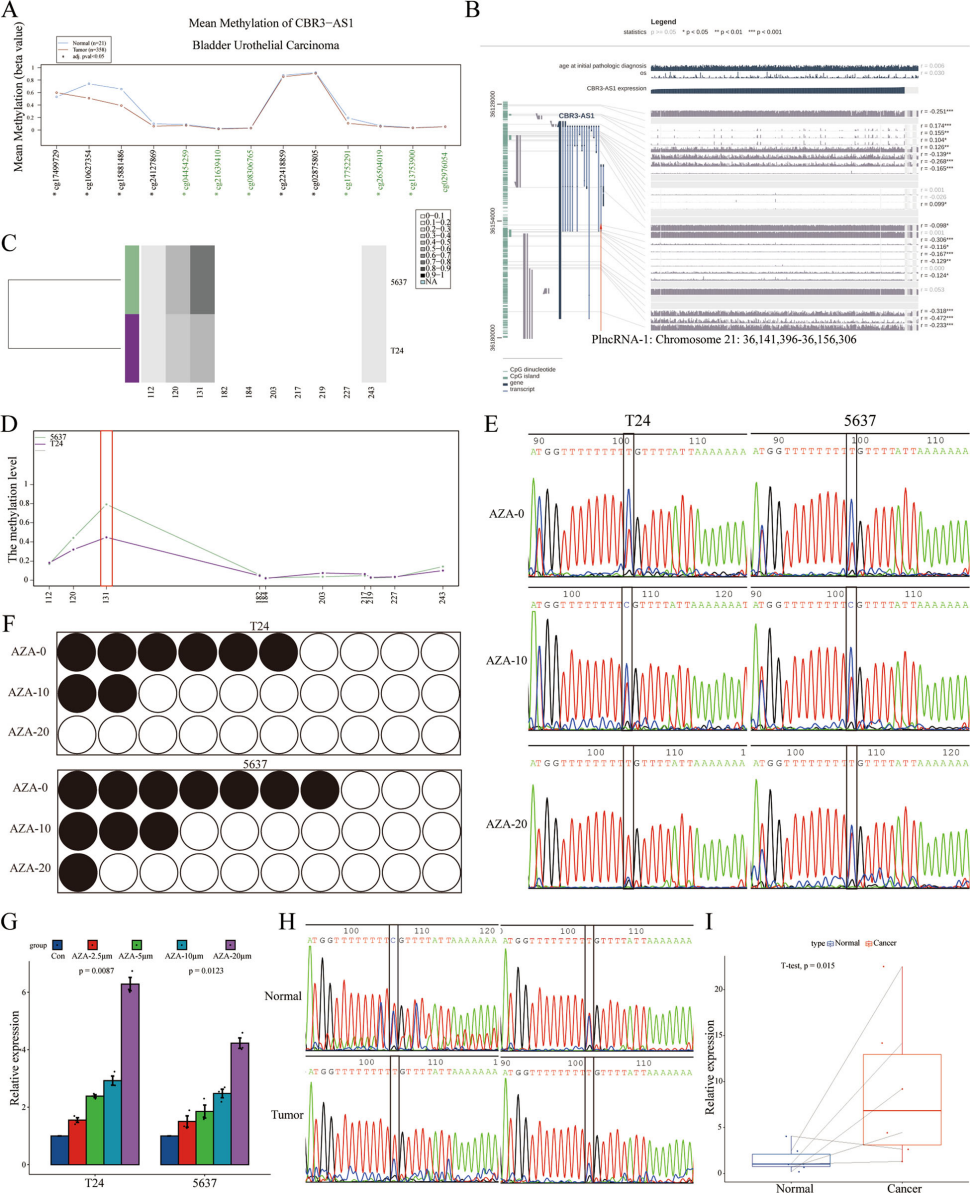
Methylation level of PlncRNA-1 promoter. **A** Wander tool analysis for the degree of PlncRNA-1 promoter methylation in BC tissues. **B** MEXPRESS tool analysis for the relationship between the degree of PlncRNA-1 promoter methylation and its expression level in BC. **C**, **D** BSAS analysis for the degree of PlncRNA-1 promoter region methylation. **E**, **F** The effect of AZA treatment on the methylation level of PlncRNA-1 promoter at position 131. The red peak represents the base T, which is unmethylated. The blue peak represents the base C, which is methylated. Black dots represent methylation, whereas white ones represent unmethylation. **G** The expression level of PlncRNA-1 after treatment of BC cells with varied AZA concentrations. **H** Degree of PlncRNA-1 promoter region methylation in BC tissues. **I** The expression level of PlncRNA-1 in six pairs of normal and BC tissues.

PlncRNA-1 is highly expressed in bladder cancer tissues, and the level of expression corresponds with the pathological stage of cancer. PlncRNA-1 could mediate various parameters of tumor cells both in vivo and in vitro. Therefore, to further explore factors mediating the high expression of PlncRNA-1 in bladder cancer tissues, we analyzed the methylation level of the PlncRNA-1 promoter region using Wanderer, an intuitive web tool for real-time access and visualization of gene expression and DNA methylation profiles. As shown in Fig. 4A, compared to normal tissues, BCs exhibited lower methylation levels of the PlncRNA-1 promoter region. MEXPRESS further revealed a negative correlation between the degree of methylation in the PlncRNA-1 promoter region and the level of PlncRNA-1 expression (Fig. 4B). Since PlncRNA-1 was differentially expressed in T24 and 5637 BC cells, we used BSAS to analyze the transcription start site, 2000 bp upstream, and 1000 bp downstream of PlncRNA-1 gene in these cell lines. As shown in Fig. 4C, D, the promoter region of PlncRNA-1 was found to be methylated at position 131 of PlncRNA-1 promoter (located at position 36157603 of chromosome 21). Notably, the methylation levels were relatively high in both cell lines. The difference in the expression levels of PlncRNA-1 between both cell lines at position 131 of the PlncRNA-1 promoter was significant. Therefore, the focus was on the position 131 sites of the PlncRNA-1 promoter.

Different concentrations of AZA, a DNA methylation inhibitor, were used to impair the methylation of PlncRNA-1 to varying degrees. After DNA extraction and sequencing, it was found that the concentration of AZA was directly proportional to the degree of methylation at position 131 of the PlncRNA-1 promoter region of T24 and 5637 cells (Fig. 4E). Sequence analysis of 10 random MSP amplicons revealed comparable findings (T24: 60% for AZA—0 μm, 20% for AZA—10 μm and 0% for AZA—20 μm; 5637: 70% for AZA—0 μm, 30% for AZA—10 μm, and 10% for AZA—20 μm) (Fig. 4F). Therefore, AZA can effectively modulate the degree of PlncRNA-1 promoter methylation. Furthermore, qPCR tests were performed to assess the expression level of PlncRNA-1 after treatment of T24 and 5637 cells using different AZA concentrations. As shown in Fig. 4G, the concentration of AZA was directly proportional to the expression level of PlncRNA-1. Finally, BSP was performed on 6 pairs of randomly selected pairs of normal and BC tissues with regard to position 131 of PlncRNA-1 promoter. Compared to the normal tissues, the degree of BC methylation at position 31 of the PlncRNA-1 promoter region was lower than that of normal tissues (Fig. 4H). In addition, the expression of PlncRNA-1 was higher in BCs than in normal tissues (Fig. 4I). This underlines the intriguing role of PlncRNA-1 promoter methylation at position 131 and the expression of PlncRNA-1. Hypomethylation of the PlncRNA-1 promoter at position 131 in the bladder cancer tissue mediated the high expression of PlncRNA-1.

### PlncRNA-1 regulates the expression of smad3

How did PlncRNA-1 regulate the proliferation and invasion of BC cells? PlncRNA-1 was first discovered in prostate cancer, and we performed microarray analysis in prostate cancer after interfering with the expression of PlncRNA-1. Gene chip analysis was performed after the expression of PlncRNA-1 in prostate cancer cells was interfered with. This manipulation was found to significantly impair smad3 expression in these cells. This study revealed that the interference of PlncRNA-1 expression significantly inhibits the migration and invasion of bladder cancer cells and smad3 was related to tumor migration and invasion. Combining the above two points, we take smad3 as the object of research. However, are these functions mediated by smad3?

**Fig. 5:**
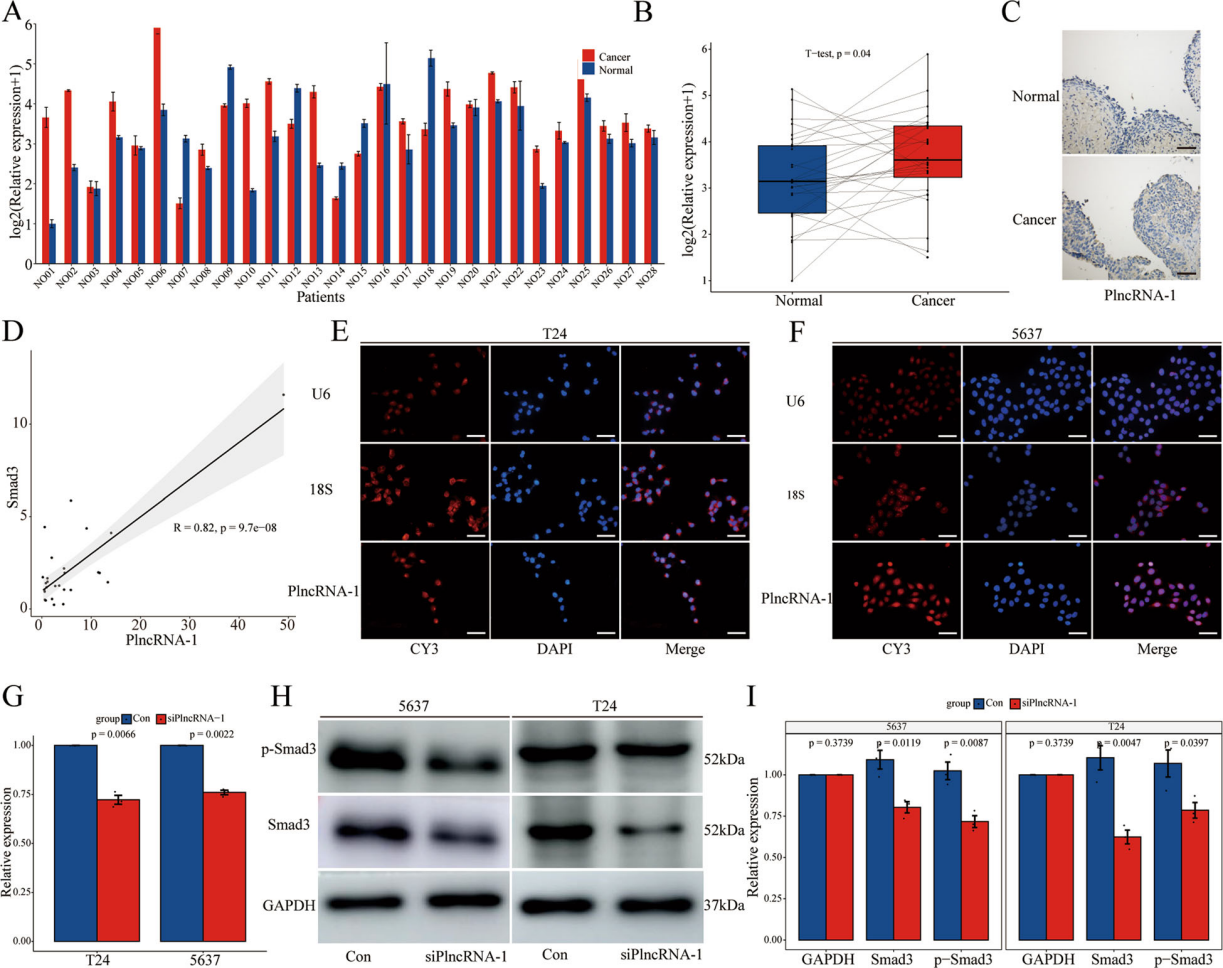
Regulation of smad3 expression by PlncRNA-1. **A**, **B** The expression level of smad3 in 28 pairs of normal and BC tissues. **C** The associations between PlncRNA-1 expression levels and smad3 in BC tissues. **D**–**F** RNA-FISH images for subcellular localization of PlncRNA-1 in BC cells (Scale bar, 50 μm). **G** qPCR analysis for the expression level of smad3 in PlncRNA-1 silent BC. **H**, **I** Western blot for the expression level of smad3 in PlncRNA-1 silent BC cells.

As shown in Fig. 5A–C, the expression level of smad3 in BC tissues revealed that smad3 was significantly overexpressed in 75.00% (21/28) of bladder cancer tissues when compared to the matched normal tissues. A positive correlation was subsequently established between the expression of PlncRNA-1 and smad3 in BC tissues (*R* = 0.82, *p* = 9.7e−08) (Fig. 5D). The intracellular distribution of PlncRNA-1 was evaluated using the RNA FISH assay. Figure 5E, F shows that PlncRNA-1 is majorly distributed in the nucleus, with part of its elements in the cytoplasm. The Human Protein Atlas (https://www.proteinatlas.org/), revealed a similar distribution of smad3. Because PlncRNA-1 and smad3 exhibited a positive correlation regarding their expression, we further explored the relationships between the two molecules. We found that PlncRNA-1 interference in T24 and 5637 BC cells significantly modulated the mRNA expression level of smad3 (Fig. 5G). Western blot further validated these findings, in which both smad3 protein and it’s phosphorylated (p-smad3) form was underexpressed in PlncRNA-1 modulated cells (Fig. 5H, I). Therefore, we postulate that PlncRNA-1 regulates the expression of both smad3 and p-smad3 in BC.

### PlncRNA-1 regulates the expression of miR-136

**Fig. 6:**
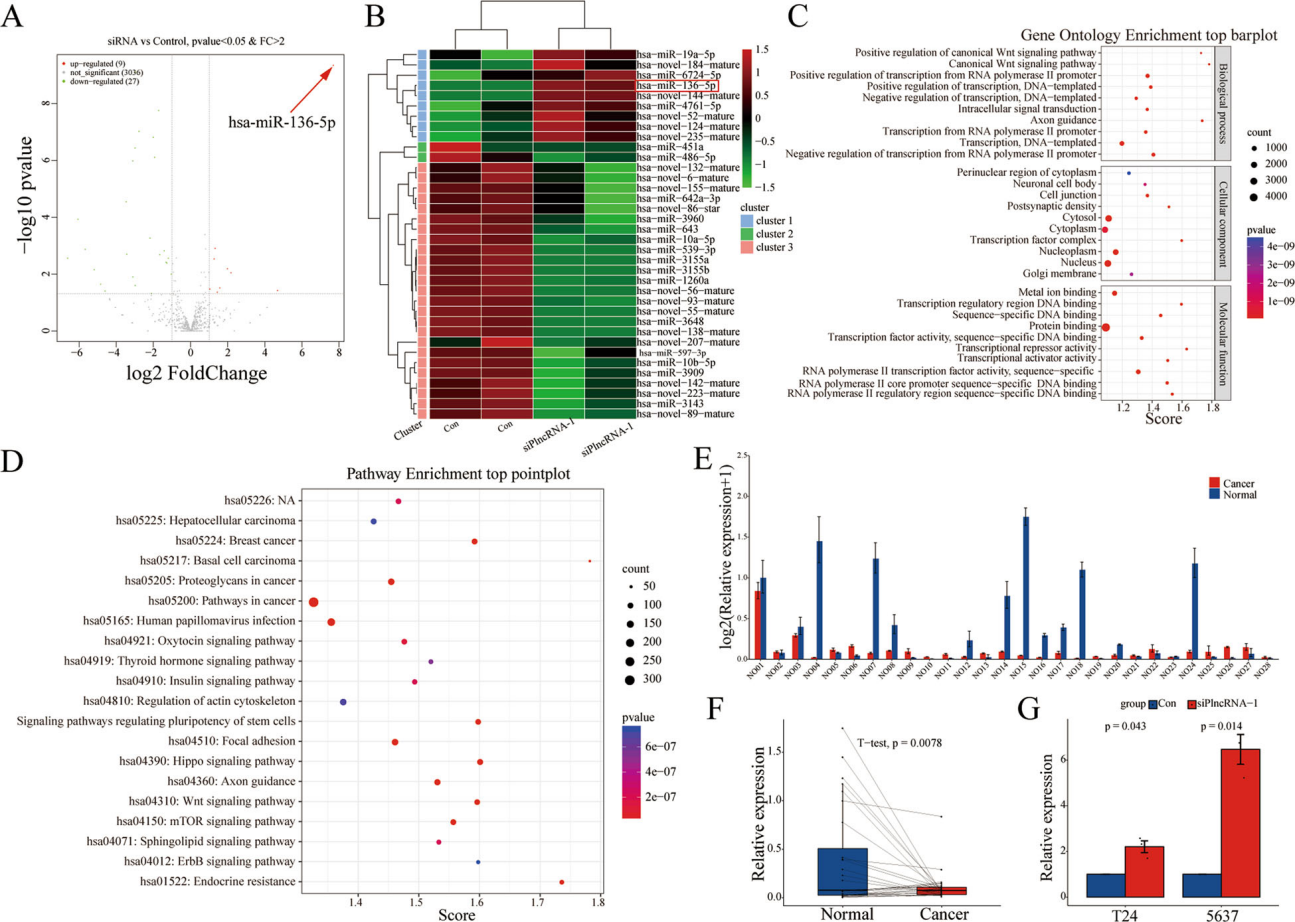
The regulatory effect of PlncRNA-1 on the expression of miR-136. **A** Volcano plots for the differential expression of miRNA between siPlncRNA-1 and the control group (Red—high expression; blue—low expression). **B** Heatmap for the differential expression of miRNA between siPlncRNA-1 and control group (Red indicates high expression; blue indicates low expression). **C** GO analysis for miRNA-predicted target genes. **D** KEGG analysis for miRNA-predicted target genes. **E**, **F** The expression of miR-136 in 28 pairs of normal and BC tissues. **G** qPCR analysis for the expression of miR-136 in BC cells after PlncRNA-1 silencing.

One of the common mechanisms by which long non-coding RNAs mediate their regulatory functions is through mRNA sponging mediated by mimicking ceRNA. Therefore, sequenced miRNA after the interference of PlncRNA-1 in 5637 BC cell lines. Thirty-six miRNAs were found to be differentially expressed, of which 27 were down-regulated while 9 were up-regulated (Fig. 6A, B). We then predicted the target genes for differentially expressed miRNAs using the Miranda tool. Thereafter, we performed GO analysis using the ‘clusterProfiler’ software (including biological process, cellular component, molecular function). KEGG analysis of the target genes was also performed to evaluate the expression profile of PlncRNA-1. Figure 6C shows that PlncRNA-1 modulates several biological processes, such as transcription, canonical Wnt signaling, and intracellular signal transduction. Cellular targets for PlncRNA-1-mediated differently expressed miRNAs include the nucleus, cytoplasm, and cytosol (Fig. 6C). Moreover, molecular functions mediated by PlncRNA-1 target genes include protein and DNA binding as well as transcriptional activity (Fig. 6C). The main KEGG processes mediated by PlncRNA-1 target genes were found to include multiple cancer surveillance pathways, focal adhesion, regulation of actin cytoskeleton, mTOR signaling pathway, Wnt signaling pathway, and transcriptional activity (Fig. 6D). The has-miR-136-5p, associated with tumor migration and invasion, was the most differentially expressed miRNA. Therefore, we explored the roles of miR-136 in follow-up research.

qPCR analysis revealed that compared to matched normal tissues, the expression of miR-136 was significantly suppressed in BC tissues (*p* = 0.0078) (Fig. 6E, F). Accordingly, interference with PlncRNA-1 expression in T24 and 5637 cancer cells significantly upregulated the expression of miR-136. Therefore, PlncRNA-1 can inhibit the expression of miR-136 in bladder cancer.

### PlncRNA-1 mimics ceRNA in the regulation of smad3 expression by binding to miR-136

**Fig. 7:**
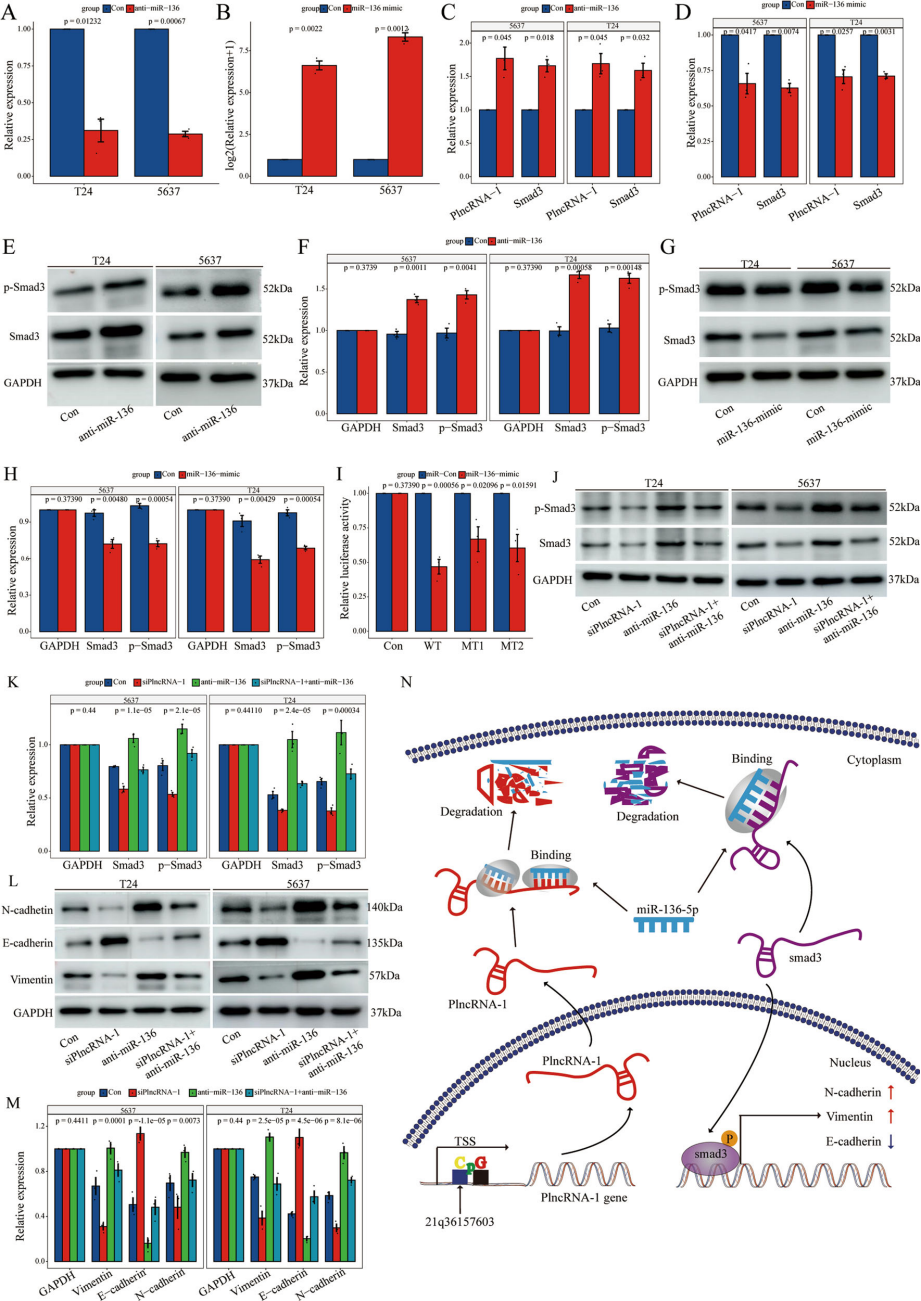
The ceRNA regulatory property of PlncRNA-1 against the expression of smad3 by miR-136 binding. **A** qPCR test for the transfection efficiency of anti-miR-136 in T24 and 5637 cells. **B** qPCR assay for the transfection efficiency of miR-136 mimic in T24 and 5637 cells. **C** qPCR assay for the expression level of PlncRNA-1 and smad3 in BC cells after miR-136 silencing. **D** qPCR assay for the expression level of PlncRNA-1 and smad3 in BC cells overexpressing miR-136. **E**, **F** Western blot for the expression level of PlncRNA-1 and smad3 in BC cells after miR-136 silencing. **G**, **H** Western blot assay for the expression level of PlncRNA-1 and smad3 in BC cells overexpressing miR-136. **I** Luciferase reporter assay for the association between PlncRNA-1 and miR-136. **J**, **K** Rescue experiment shows the relative expression level of smad3 protein in different groups. **L**, **M** Rescue experiment shows the relative expression level of EMT marker protein levels in different groups. **N** Schematic diagram of the PlncRNA-1/miR-136/smad3 axis contributing to the progression of BC. “↑” indicates upregulation, “↓” indicates downregulation, “→” indicates promotion, and “CpG” indicates CpG island. TSS transcription start site.

We have already established the regulatory effect of PlncRNA-1 on the expression of miR-136 and smad3. Therefore, we explored the relationship between PlncRNA-1, miR-136, and smad3. First, anti-miR-136, an RNA inhibitor, and miR-136-mimic were transfected into T24 and 5637 cancer cells. Interference and overexpression efficiencies were validated using qPCR. Compared to the control group, the expression of miR-136 in the anti-miR-136 group was significantly inhibited, converse to the miR-136-mimic that exhibited significantly upregulated miR-136 expression (Fig. 7A, B). These findings show that modulation of miR-136 expression significantly up-regulates the expression of PlncRNA-1 mRNA and smad3 mRNAs (Fig. 7C, D). Western blot further validated the overexpression of smad3 and p-smad3 proteins after modulation of miR-136 expression. Dual-luciferase reporter gene tests including the WT of the PlncRNA-1 transcript, MT1, and MT2 were also performed to evaluate the interaction between PlncRNA-1 and miR-136. Luciferase activity was found to be significantly suppressed in the PlncRNA-1 wild-type group. Comparable findings were reported in PlncRNA-1 MT1 and PlncRNA-1 MT2 (Fig. 7I), implying that miR-136 can directly attach to the two PlncRNA-1-binding sites.

Finally, rescue experiments were performed to explore the effect of PlncRNA-1 on the expression of smad3 through miR-136. PlncRNA-1 interference induced changes in the expression of smad3, p-smad3, and EMT protein marker were rescued by anti-miR-136 (Fig. 7J–M). Therefore, PlncRNA-1 exerts its regulatory role through the miR-136/smad3 axis. The specific regulatory mechanisms of PlncRNA-1 on smad3 are illustrated in Fig. 7N.

## Discussion

Bladder cancer is one of the most common malignant urothelial tumors worldwide. The incidences and recurrence risks of the tumor are steadily increasing. Despite advances in the current clinical treatments such as surgery, radiotherapy, and chemotherapy, the overall survival time of bladder cancer has generally remained the same^34–38^. This underlines the urgent need for new early diagnostic markers, as well as more effective and safer therapeutic options for BC. The discovery of a high number of noncoding RNA transcripts in the human genome has greatly changed our understanding of many diseases, including cancer. In tumors, the degree of lncRNA dysregulation corresponds with the disease stage and maybe an independent prognostic factor for cancer patients^39–41^. Overexpressed lncRNA SOX2OT in bladder cancer is positively correlated with a high histological grade, advanced TNM stage, and poor prognosis. Mechanistically, SOX2OT upregulates SOX2 expression by sponging miR-200c^10^. In addition, many lncRNAs such as CASC9^42^, SNHG1^43^, TUG1^44^, and NBAT1^45^ are associated with the occurrence and development of bladder cancer.

PlncRNA-1 is a new lncRNA oncogene that is located on human chromosome 21^15^. Several studies have shown that PlncRNA-1 modulates several pathways in multiple human diseases such as prostate cancer^15,16,21,29^, esophageal cancer^25^, hepatocellular carcinoma^18^, gastric cancer^17^, septic acute kidney injury^19^, colorectal cancer^20,24^, breast cancer^22^, retinoblastoma^26^, and glioma^27^. However, the clinical significance and biological functions of PlncRNA-1 in bladder cancer are largely unknown. In this study, qPCR for the expression of PlncRNA-1 in 28 tissue pairs revealed significant elevated PlncRNA-1 expression levels in BC tissues. Moreover, the degree of PlncRNA-1 expression corresponded with tumor invasion, T stage, age, and a number of BC tumors. PlncRNA-1 was also highly expressed in MIBC than in NMIBC tissues. A similar trend was observed in BC stage T3–T4 and T1–T2. In order to compare the predictive value of the expression level of PlncRNA-1 on the clinical characteristics, we compared the proportions of subgroups in the high or low PlncRNA-1 expression group. In the low PlncRNA-1 expression group, T1–T2 stage accounted for 100% while the MIBC stage accounted for 42.86%. In the high expression group, T1–T2 stage accounted for 57.14% while the MIBC stage accounted for 85.71%. If the expression level of PlncRNA-1 was low in BC patients, then the probability of BC patients in T1–T2 stages was 100%, and the probability of BC patients in the MIBC stage was 42.86%. If the expression level of PlncRNA-1 was high in BC patients, then the probability of BC patients in the T1–T2 stage was 57.14%, and the probability of BC patients in the MIBC stage was 85.71%. Of course, a large sample is needed in the future to further verify the predictive ability of PlncRNA-1. Therefore, PlncRNA-1 has a strong clinical value. Function analysis further showed that inhibition of PlncRNA-1 expression significantly suppressed the proliferation, migration, and invasion of bladder cancer cells in vitro. In addition, the inhibition significantly enhanced the tumorigenicity of bladder cancer cells in vivo. This underlines the potential clinicopathological role of PlncRNA-1 in BC, in particular its diagnostic as well as therapeutic values.

In general, PlncRNA-1 is over-expressed in bladder cancer cells, promoting their proliferation and invasion. Therefore, PlncRNA-1 typifies an oncogene. The functional properties of PlncRNA-1 are mediated by DNA methylation in cancer cells^46^. DNA methylation is a cancer hallmark^47^. The overall genome hypomethylation in cancer cells destabilizes genomic stability and induces the re-expression of undesirable silent genes^47,48^. Using Wanderer and MEXPRESS online tools, we found multiple hypomethylations in the PlncRNA-1 promoter region. The level of methylation was negatively correlated with PlncRNA-1 expression. In particular, methylation occurred at position 131 positions of the PlncRNA-1 promoter region (located at the 36157603 positions of chromosome 21). Compared to the normal tissues, BC tissues exhibited hypomethylation of the PlncRNA-1 promoter at position 131, with PlncRNA-1 expression being upregulated in BC tissues. Inhibiting methylation of the PlncRNA-1 promoter at position 131 suppresses PlncRNA-1 expression. There, it is a potential therapeutic target for bladder cancer.

LncRNA exerts its functions in multiple ways, with the ceRNA hypothesis attracting considerable attention. This hypothesis suggests that by sharing a common MRE, lncRNA can act as a miRNA “sponge”, thereby regulating downstream target genes^13,49,50^. The ceRNA network can link protein-coding and noncoding mRNAs, effectively modulating the post-transcription of the mRNAs. Mechanistically, we postulated that PlncRNA-1 acts as ceRNA through miRNA sponging. First, we found that PlncRNA-1 regulates the expression of smad3. In prostate cancer, gene chip sequencing revealed the downregulation of smad3 expression after PlncRNA-1 interference. In bladder cancer, PlncRNA-1 regulates cell migration and invasion, whereas smad3 promotes the EMT of tumor cells^51,52^. Furthermore, FISH revealed that PlncRNA-1 is mainly located in the nucleus and partly in the cytoplasm. Intriguingly, the distribution of smad3 in the cell is comparable to that of PlncRNA-1. Therefore, we hypothesized that PlncRNA-1 regulation of proliferation, migration, and invasion of bladder cancer cells is mediated by smad3. In this study, PlncRNA-1 interference decreased the expression of smad3 and psmad3, strengthening our hypothesis. Subsequently, miRNA-seq revealed significant expression changes in 36 miRNAs after the interference of PlncRNA-1 expression in BC tissues. Among them, has-miR-136-5p exhibited the most significant changes. Multiple studies have documented the EMT role of miR-136 in several tumors, including colon^53^, gastric^54,55^, breast^56^, and renal cell carcinoma^57^. StarBase online prediction tool showed that miR-136 and smad3 have a common binding site. Studies have also reported that miR-136 inhibits the epithelial-mesenchymal transition and metastatic properties of lung adenocarcinoma by targeting smad2 and smad3^33,58^. Therefore, we hypothesized that PlncRNA-1 regulates the proliferation, migration, and invasion of bladder cancer cells through miR-136. However, PlncRNA-1 interference unregulated the expression of miR-136, validating our hypothesis. Finally, we performed dual luciferase and rescue experiments in order to determine whether PlncRNA-1 mimics ceRNA in regulating smad3 expression, particularly by binding miR-136. After interference or overexpression of miR-136, PlncRNA-1, and smad3 exhibited significant corresponding changes, suggesting that miR-136 can also regulate the expression of PlncRNA-1 and smad3. Base sequence analysis of both PlncRNA-1 and miR-136 revealed that the two polymers have two binding sites. Thus, the addition of miR-136 mimic to bladder cancer cells reduced the fluorescence intensity of the wild-type PlncRNA-1, similar to the fluorescence intensity of mutant site 1 and the mutant site 2 groups, albeit less than the wild-type group, implying that PlncRNA-1 and miR-136 can directly bind. In addition, based on rescue experiments, silencing miR-136 partially impaired the expression of smad3 and EMT marker proteins mediated by PlncRNA-1. Therefore, PlncRNA-1, as a ceRNA for miR-136, promotes the expression of smad3, thereby regulating the occurrence and development of bladder cancer.

Our findings notwithstanding, this study had several limitations. LncRNA participates in chromatin looping, nuclear body formation, and function, molecular decoys, mRNA splicing, inhibition of translation, and miRNAs sponging or competition for miRNA-binding sites on mRNA^59,60^. In particular, lncRNA can influence transcription, splicing, translation, export, import, and stability of mRNA^61^ In this study, we only studied the effect of PlncRNA-1 on the regulation of proliferation and invasion of bladder cancer through smad3, overlooking other regulatory mechanisms, such as those mediated by lncRNA.

In conclusion, we have demonstrated PlncRNA-1 is significantly upregulated in BC tissues, and its expression positively correlates with tumor invasion, T stage, age, and tumor numbers. The expression level of PlncRNA-1 can be used to predict the degree of tumor invasion and T stage of BC to a certain extent. These effects imply the carcinogenic effects of PlncRNA-1 and its important clinical value in bladder cancer. Both in vivo and in vitro studies have shown that PlncRNA-1 can modulate the proliferation, migration, and invasion of BCs. In addition, hypomethylation of the PlncRNA-1 promoter at position 131 enhances the expression of PlncRNA-1. In conclusion, PlncRNA-1 regulates the proliferation and invasion of bladder cancer by miR-136 sponging and by increasing the expression of smad3. Our research provides new insights into the post-transcriptional regulation mechanism involved in PlncRNA-1. The PlncRNA-1/miR-136-5p/smad3 axis is a potential diagnostic and therapeutic target for BC.
